# Acceptability of a digital return-to-work intervention for common mental disorders: a qualitative study on service user perspectives

**DOI:** 10.1186/s12888-021-03386-w

**Published:** 2021-08-03

**Authors:** Patrik Engdahl, Petra Svedberg, Ulrika Bejerholm

**Affiliations:** 1 Lund University, Department of Health Sciences/Mental Health, Activity and Participation, Lund University, P.O BOX 157, SE-22100 Lund, Sweden; 2 Halmstad University, School of Health and Welfare, Halmstad, Sweden

**Keywords:** Return to work, Mental health, Depression, Anxiety, Digital solution, Vocational rehabilitation

## Abstract

**Background:**

There is an evident discrepancy between need and provision of evidence-based return-to-work (RTW) interventions in existing mental health services. Online dissemination of evidence-based interventions is presumed to reduce this gap. However, there is almost no knowledge available on perceived acceptability of digital RTW interventions among service users, which are factors that might influence the development and implementation of future interventions. The aim of this study was to develop knowledge of service user acceptability of mWorks, a proposed digital RTW solution.

**Methods:**

Participants (*n* = 18) with experience of common mental disorder and sick leave were recruited with a purposive snowball sampling method. Semi-structured interviews (*n* = 12) and one focus group interview (*n* = 6) were conducted. A deductive thematic analysis was performed according to the Theoretical Framework of Acceptability.

**Results:**

Digital RTW interventions were perceived as acceptable and aligned with participant value. Participants expressed positive attitudes toward having access to support, regardless of time and place. A certain ambiguity between a decline in social interactions and opportunities to RTW in a safe space was reported. Participants were confident in their ability to use digital RTW solutions, but reported the need to reduce stressful elements of using smartphones. Overly demanding digital solutions, i.e. ones requiring high cognitive effort, were described as burdensome.

**Conclusions:**

For digital RTW solutions to be acceptable, they need to complement traditional services by providing accessible and person-centred support throughout the RTW process. They should be designed to reduce the need for cognitive effort. Future research should explore how to balance user autonomy with other support components in digital interventions.

**Supplementary Information:**

The online version contains supplementary material available at 10.1186/s12888-021-03386-w.

## Background

Common mental disorders (CMD) such as depression and anxiety are increasing globally and constitute one of the most common causes of reduced health [1]. These illnesses are associated with substantial personal burdens, i.e. negative impact on well-being, lessened financial security, and risk of social isolation. In addition, mental illness contributes to a substantial societal cost in terms of sick leave, health care, and lost productivity estimated to cost €600 billion annually in Europe alone [2]. Many individuals with CMD are on long-term sick leave [3]. The societal cost of sick leave due to CMD is double that of individuals with physical conditions [4].

Few effective return-to-work (RTW) interventions exist for the target group of individuals with CMD [5]. Traditionally, RTW support is fragmented and follows a step-by-step approach, a medical model in which single interventions in health services, e.g. medication and cognitive behavioural therapy, constitute the first steps and there is little connection to other RTW welfare services or the workplace [5–9]. The Swedish welfare system is highly sectored and the responsibility and commitments of a service user’s RTW process are scattered among Health Services, the Social Insurance Agency (SIA), the Public Employment Service (PES), and Social Services [7, 10]. Service and knowledge gaps create barriers to RTW and prolong periods of sick leave [6, 11–13]. In response to a dearth of effective RTW interventions, evidence-based supported employment (SE) for persons with severe mental disorders (SMD) [14] has been adapted with cognitive behavioural therapy (CBT) strategies to better fit the support needs of persons with CMD. This kind of intervention where SE and CBT strategies are integrated has been shown to be more effective than traditional services on RTW among persons with CMD in one Norwegian and one Swedish pragmatic, parallel randomized controlled trials [15–17]. However, recent implementation research highlights the discrepancy between the need and access to effective interventions [18]. This dilemma is largely caused by two conflicting RTW paradigms, one that fosters a person-centred, strength-based, and recovery-oriented SE approach, and one that holds traditional standards and focuses on diagnosis, functional disability, and activity limitations [7, 17, 18]. In addition, staff and employers engaged in the traditional approach have shown to have low mental health literacy, adding to the knowledge and service gap [13, 19, 20]. Digital solutions have sparked a new hope of making RTW interventions accessible to a broad audience [21]. Our aim was to develop a digital RTW intervention that covered the features of adapted SE intervention that include CBT strategies [15, 22]. Online delivery through smartphones may improve access to RTW interventions and play a role in reducing the service and knowledge gap.

As a precondition to evaluate effectiveness of interventions, the Medical Research Council has provided guidance on the need to test and refine such interventions to assure they are acceptable [23]. Acceptability is a multi-factored construct that reflects the extent to which people perceive an intervention to be appropriate, i.e., anticipated or experiential cognitive and emotional responses to an intervention. Assessment of acceptability can take place before, during or after the intervention experience [24]. However, there is an absence of a clear and shared framework of acceptability, which has led to an insufficiently robust research corpus. To remedy this, Sekhon and colleagues [24] provided the Theoretical Framework of Acceptability (TFA), the first systematic approach to developing a common understanding of acceptability. Researchers have recently begun to apply the TFA to evaluate acceptability in different stages of complex interventions, including development, evaluation, and implementation. For example, the TFA was applied in evaluation of the experiences of community pharmacists working in a men’s mental health program that helped to identify acceptability issues and inform changes in program design [25].

Previous research on acceptability in relation to CBT delivery formats shows a conflicting picture of whether face-to-face or digital interventions, with or without human support, are preferable. These inconsistent research results are likely due to comparisons of different formats [26, 27]. Two meta-analysis and a review showed that digital interventions with human support yield better outcomes than interventions without human support [26, 28, 29]. Lower acceptability would therefore be expected for digital interventions in unguided internet CBT (iCBT). However, this was surprisingly not found in a meta-analysis of delivery formats. One explanation may be that acceptability was operationalised as study dropouts, and not as experiential responses to an intervention [26]. Inconsistencies in the current research corpus suggest a need to investigate acceptable delivery formats according to a standardized framework and the role of professionals when determining whether a digital RTW intervention will be successful.

Poor engagement of service users in digital solutions in primary care contexts, as well as slow dissemination, suggest other acceptability barriers [30, 31]. Acceptability has been explored during transformation of evidence-based interventions (e.g., CBT) to digital solutions (e.g., iCBT). A qualitative meta-synthesis concluded that acceptability relies on the sensitivity of the digital intervention to individual needs and preferences [32]. In a feasibility study, evaluating the acceptability of a digital solution that aimed to decrease depressive symptoms and increase well-being at the workplace, it was reported that engagement issues constituted an acceptability barrier. This was attributed to the fluctuation of service users’ mental health, and that they did not have enough time for app usage which resulted in disengagement [33]. However, little is known about the acceptability of digital RTW interventions from the perspectives of service users. Thus, there was a need to understand prospective acceptability, i.e., anticipated acceptability of mWorks, a proposed digital RTW intervention to be delivered by RTW professionals and used by persons on sick leave due to CMD throughout the RTW process. This understanding will serve to modify aspects prior to implementation, and thus inform the content of the proposed intervention.

While some research lessen the importance of digital intervention characteristics, other highlights the need to address these characteristics for service user acceptability. The latter are associated with emotional state, attitude, and the severity of depression, each of which affects service user acceptability of an intervention [24, 34]. Insights from a previous acceptability study on digital positive psychology intervention for persons with CMD add that factor such as a persuasive design, easy accessibility, a credible reputation, and not requiring too much effort for interaction, personality and symptom severity were important to consider when creating an acceptable digital solution [34]. Symptom severity is associated with low levels of engagement, mediated by decreased levels of motivation and interest in previously enjoyable tasks [35]. Therefore, it is vital to establish how digital interventions such as mWorks can be designed to meet potentially decreased engagement levels of service users with CMD.

A lack of conclusive knowledge hampers the ability to design acceptable digital interventions for this target group. Qualitative methods are well suited to investigate anticipated acceptability of the intended audience [24, 34, 36]. No research has previously examined perceived acceptability of a digital RTW intervention with a standardized framework of acceptability. Thus, by conducting a qualitative thematic analysis we aimed to decrease the knowledge gap and increase the understanding of service user acceptability of mWorks, a proposed digital return-to-work solution for persons with experience of CMD and sick leave using the TFA.

## Methods

### Design

A qualitative research design with a deductive thematic approach [37] was used in order to analyse participant perceptions of acceptability of mWorks. A top-down thematic analysis method was chosen because it tends to generate detailed information about specific aspects of the data, in this instance, information related to the seven acceptability attributes of the TFA [20]. This is in contrast to a bottom-up thematic analysis which tends to yield richer descriptions from the entire data corpus [37].

This study is part of a larger project in the southern region of Sweden with the aim or developing and evaluating a digital RTW intervention, mWorks, for persons with CMD [22]. It is in accordance with the 2008 revision of the Helsinki Declaration has been approved by the Ethical Review Board in Lund, Reg. No 2017/324. This study was guided by consolidated criteria for reporting qualitative research, COREQ [38].

### Recruitment and participants

Inclusion criteria included being of working age, 18–65 years, having current or lived experience of sick leave and the RTW process, self-reported diagnosis of a CMD, (i.e., depression, including depressive episodes inherent in bipolar disorder and/or anxiety disorder) and able to communicate in Swedish.

A purposeful snowball sampling method was utilized where initial participants nominate other potential participants using their network [39]. This recruitment method enabled us to find information-rich participants that otherwise are difficult for researchers to access, who belong to a vulnerable group in various care or RTW support programs for persons with CMD [40]. Initially, the first author (PE) contacted four previously known mental health and RTW professionals who had regular contact with potential participants during the course of their daily work at social services, Fountain House clubhouses (non-profit mental health service where members are provided with opportunities for RTW support), primary care, and mental health services. The professionals were asked to nominate individuals who met the inclusion criteria. The nominated individuals were contacted via email or face-to-face by the professionals and asked to participate. They received oral and written information about the study. To verify the inclusion criteria, nominated participants were asked by the first author (phone, email) if they recently had been sick-listed due to CMD (according to medical certificate for sick leave), if they currently were involved in a RTW process, or had prior experience of being on sick-leave due to CMD and involved in a RTW process. The initial two individuals agreed to participate. These two participants then nominated additional individuals, and asked if they wanted to participate. If the additional individuals were interested in participating, they were contacted by the first author (PE) and further informed about the study. All participants gave written informed consent prior to the interview.

### Data collection

Individual (*n* = 12) and focus-group (*n* = 6) interviews were conducted between April 2017 and January 2018. The individual interviews were intended to generate a broad range of topics. The focus group interview aimed to reveal additional insights about more sensitive and personal viewpoints. These revelations are more frequently occurring in a focus group context where participants from a homogeneous group can explore their group identity, challenge aspects inherent to their subculture, and thereby exposing aspects that ordinarily are out of reach in an individual interview context [41]. The interviews were semi-structured [42] and focused on generation of information about participant experiences, needs and preferences concerning the mWorks intervention. Prior to the interviews, participants had accessed verbal and written information about the purpose and design in connection to informed consent. In addition, brief and standardized verbal information about the project and proposed digital RTW solution was written at the top of the interview guide to be easily introduced. The interview guide was the same for both types of interviews and derived from a similar study that aimed to develop a digital service for childhood cancer survivors within a health service context [43]. The interview guide was adjusted to fit the current target group by addition of probing questions regarding the RTW context. According to the preferences of the participants, interviews took place at participant homes or at the university research facilities (Lund University). The first author (PE) conducted most individual interviews, while the last author (UB) conducted the first individual interview and moderated the focus group interview with an assisting researcher who took field notes and posed probing questions when needed. Each interview was audio-recorded and supplemented with field notes to capture additional observations that added meaning and understanding to the interview. Individual interviews lasted approximately 30 to 45 min, and the focus group interview lasted about 60 min. To protect participant confidentiality, each transcript was stripped of identifiable details, assigned an anonymous code, and stored securely.

### Data analysis

**Fig. 1:**
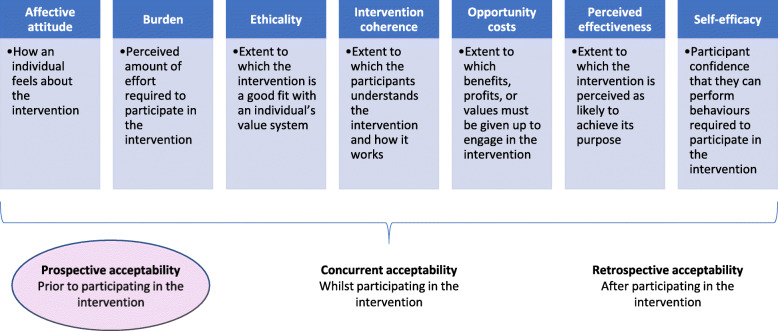
Theoretical Framework of Acceptability (TFA), developed by Sekhon and colleagues. TFA is a multi-faceted framework that reflects the extent to which people delivering or receiving a healthcare intervention consider it to be appropriate, based on anticipated or experiential cognitive and emotional responses to the interventions [24]

The recorded interview material was transcribed verbatim. The material was subjected to a “top-down” thematic analysis [37] and the themes were driven by a theoretical framework of acceptability (TFA) [24]. The framework entails seven constructs (Fig. 1) and can be used to understand how people consider a healthcare intervention to be appropriate, based on expected or experienced cognitive and emotional responses to an intervention [24]. This could be done before (prospective acceptability), whilst (concurrent acceptability) or after (retrospective acceptability) participating in an intervention. In our study we investigate prospective acceptability.

The analysis procedure initially involved reading through the field notes and transcripts. A software tool specifically developed to analyse qualitative data, Open-Code version 4.03, was used to organize and gain an overview of the data. Content from the transcripts was identified that corresponded with the acceptability attributes and initial coding into themes was performed by the first author (PE). The themes were then collapsed to smaller components to clarify distinctions in the material. Next, UB scrutinized the first author’s (PE) interpretation of the data, which was an iterative process, to ensure that the interpretations of the themes were credible. Subsequently, all three authors worked together iteratively to ensure that the narrative represented the data, which helped to form consensus. For example, one participant explained how digital solutions may provide a sense of security and safety since mWorks makes the RTW support accessible. This was assigned the acceptability construct of *affective attitude* and assigned to the theme *creates a sense of safety*.

## Results

In total, eighteen individuals agreed to participate. There were twelve individual interviews and one focus-group interview with six participants. In this sample, the distribution between the genders were favourable towards men (*n* = 11) in comparison to women (*n* = 7), with a mean age of 41 years (range 25–74). Participants reported having a mean experience of CMD 5 years (range 1–40). Depression (75%) was the most frequently reported diagnosis, followed by bipolar disorder (17%) and general anxiety disorders (8%). 22% of the participants were currently on sick-leave due to CMD (*n* = 4), 22% were currently involved in a RTW process (n = 4), and 56% had prior experience of being on sick-leave due to CMD and RTW (*n* = 10). With regards to educational level, 58% reported having completed a University degree, 33% Upper secondary school, or 8% reported of nine-year compulsory school, or lower. All participants were of ethnic origin in Sweden and spoke Swedish.

The findings presented below are based on the seven constructs of the TFA. The most commonly raised construct in our analysis was burden, followed by affective attitude, ethicality, perceived effectiveness, opportunity costs, self-efficacy, and last intervention coherence (Table 1).

**Table 1:** Acceptability of a digital return-to-work solution, mWorks, based on the Theoretical Framework of Acceptability

Construct (%)	Theme
Affective attitude (18.9)	Avoids feelings of being judged Creates a sense of safety Creates a harmonious feeling
Burden (28.2)	Need for flexibility regarding feedback Motivational difficulties Cognitive strain
Ethicality (14.8)	Increase service user control Reduced clinical and deficit-oriented approach
Intervention coherence (4.9)	Counteracts evasive behaviours
Opportunity costs (11.2)	Complement to traditional RTW support, not a substitute Safe digital space to progress
Perceived effectiveness (13.3)	Involves the entire RTW process Support regardless of place and time Enables a proactive RTW process
Self-efficacy (8.4)	Confidence in using digital platform Increase stress levels Dependent on age and earlier digital experiences

### Affective attitude

Participants stated that mWorks needed to *avoid feelings of being judged.* They expressed difficulty in facing family and professionals due to being negatively judged in the context of their shortcomings and not following through on their responsibilities. This was explained as producing feelings of guilt. In order for mWorks to be acceptable, a judgmental tone needed to be avoided.

“It should not be interpreted as judgmental if you miss certain meetings and such. Well, you can get a reminder that now you have missed this and that many, but not like a red flashing app.” [Individual interview 3]

Participants felt that mWorks should not make service users feel any different from others, regardless of their mental health problem. Otherwise, such an application would be perceived as offensive and judgmental. Furthermore, participants cautioned against designing mWorks as too childish because this would come across as stigmatizing.

mWorks needed to *create a sense of safety.* Digital solutions were thought to need to be capable of delivering support regardless of time, pace and place. Knowing that they had access to support available through their mobile device provided participants with a sense of security. In contrast frustrations about the inadequacy of traditional vocational rehabilitation services in terms of continuity and long-term sustainability were described. Not knowing if support would be available when it was most needed produced a sense of insecurity. One service user expressed frustration at being abandoned without adequate RTW support during relapse.

“But three weeks later, you are back at square one anyway...they felt that you were done there, somehow.” [Individual interview 10]

Participants described the need to *create a harmonious feeling* when they signed in to mWorks for the first time. This was regarded as important to arrive in a space that were pleasant and free from annoyances. Likewise, a focus on positives instead of negatives would facilitate a harmonious atmosphere. In contrast, too many required actions, software bugs, and unresponsive tactile sensation (i.e. input delay when touching the mobile screen) could contribute to the elimination of a harmonious impression.

### Burden

Participants explained the *need for flexibility regarding feedback* to reduce the perceived burden of using a digital platform. There were contradictory statements in relation to feedback, making it clear that feedback is a double-edged sword. The use of feedback was perceived as both a facilitator and a barrier for RTW. On the one hand, feedback was a powerful tool to increase motivation through rewarding the completion of tasks, showing user progress and return-to-work trajectory. On the other hand, feedback could be burdensome, since negative patterns would become apparent and could reinforce negative emotions, thoughts and behaviours. Participants recommended that feedback should not be mandatory and should be used with caution. The suggestion was for mWorks to foster individualized options of how to use and approach feedback functions.

*Motivational difficulties* were described as an engagement barrier for mWorks. According to the participants, this was predominantly a product of depressive symptoms that may make it burdensome to engage with a proposed digital intervention. Participants anticipated that engagement with mWorks would be difficult during more severe periods of depression or anxiety. They all shared experiences of having problems with getting out of bed and wondered how they would have the energy and mental fortitude to participate in the intervention.

“It is hard enough to do things at all, because you are so terribly exhausted or depressed. So why does it make sense to do it? ... You do not see that much meaning in doing things. You do not think that it will help anyway.” [Individual interview 10]

One participant suggested the importance of providing the user with the why(s) for using mWorks, i.e., why this specific activity would be useful for the RTW process. Such understanding could provide smaller activities or subtasks with meaning.

Participants recommended that mWorks limit the *cognitive strain* required to interact with the support tool. Their depression and anxiety contributed to high cognitive strain, and this made it burdensome to interact with cognitively demanding devices. Therefore, the cognitive barrier needs to be sufficiently low that service users are able to intuitively understand mWorks in terms of use, orientation, and where to start. Ideas were elaborated to make mWorks less cognitive demanding, including limiting the initial number of actions and choices, while gradually introducing more functionality. Participants suggested that a large amount of text should be limited, and symbols and colours should be used in a systematic way to facilitate a sense of order and structure. This was assumed to make mWorks easier to use. As an example, one participant explained how different colours for different care organizations could help:

“That there may be different colours for when it is about work, when it concerns the municipality, and when it is (health) care. Then you get a brief overview... Okay, now I have some municipal meetings there, some care meetings there, and some meetings at work there.” [Individual interview 1]

### Ethicality

The participants valued *increased service user control* over the use and access to their own data and progress. They explained that no one but service users themselves should dictate how to use mWorks. Rather, autonomy regarding how much and what parts of mWorks to use was valued. Self-determined involvement was perceived to foster ownership of their RTW process. Although most participants valued the ability for service user control, one participant did not think user control was preferable. On the contrary, leaving the responsibility to the professionals was a relief. Furthermore, data generated by the service user must be secure, with access restricted to the user. Taking adequate safety measures were described as paramount in order to guarantee the safety of personal information.

“It should be very clear that it is you alone who governs this. That it is you who are the focus. It is you that this is about. So, if you don't want A, B, or C to get some information or know that you have missed these meetings ... they shouldn't be able to do that either. They shouldn't be able to go in the back way somehow. [That] you should feel safe.” [Individual interview 3]

Participants wanted to share their data with other RTW actors, under the condition that the service user had full authority to decide who would have access to their data.

A *reduced clinical and deficit-oriented approach* suited participant values. Having a clinical approach or focus was felt to place emphasis on negative aspects and problems*.*

“I think it shouldn’t be too clinical. It shouldn’t say ‘the County Council of Scania’ on it.” [Individual interview 7]

Participants wanted mWorks to promote positivity and focus on the recovery process and problem-solving. A clinical focus was felt to contribute to reinforcement of the individual’s self-stigma for being on sick-leave and having a mental illness.

### Intervention coherence

Participant understanding of mWorks indicated that the intervention *counteracts evasive behaviour.* The use of calendars and notifications were explicitly mentioned as tools to mentally prepare for daily tasks and behaviours required to progress toward RTW. Mental preparation was described as counteracting evasive behaviour since the intervention strengthened the individual by planning and strategizing about future events, such as meetings with rehabilitation actors.

“If it turns out that in two hours I’ll have to go to this meeting and I wasn’t mentally prepared, then it might be that I don’t go at all.” [Individual interview 1]

Seeking family member approval in relation to the participants and their life situation was described as a constant challenge. mWorks was perceived to legitimize their actions towards RTW, concerning their family members, or other persons in their social network. They could be transparent about what they were working on, their progress, and what rehabilitation actors were involved in the process. Participants reported that displaying where they were in their RTW process to family members provided them with a feeling of accountability, and made them more likely to follow through on their commitments.

### Opportunity costs

Participants cautioned about the potential danger of replacing human contact with a digital interaction. Therefore, in order to be acceptable, a digital intervention must be designed as *a complement to traditional RTW support, not a substitute*. Some participants valued the social interaction with rehabilitation actors. This benefit was thought less likely to occur if human interaction was replaced with digital contact. However, some individuals prefer to manage their contact with RTW actors through digital means, and it was highlighted that individual preferences should dictate the levels of human interaction. The cost of reducing human contact was compensated for by mWorks making it possible to have a *safe digital space to progress.* Participants valued a digital space where service users could process RTW related issues and progress towards RTW in a safe space, free from external stressors, in an environment of their choosing.

### Perceived effectiveness

For participants to accept mWorks as an effective RTW intervention, it must *involve the entire RTW process*. The advantage of using mWorks was the ability to gather everything related to the RTW process in one place. mWorks needed to have a holistic view of the service user and not focus blindly on the RTW outcome alone. To focus on everyday needs such as food, medication, and general well-being was also important.

Participants had confidence that digital strategies could reduce stress and anxiety. For example, internet-delivered Cognitive Behaviour Therapy (iCBT) and other digital meditation practices were mentioned as effective treatments because users can access *support regardless of place and time*. Some scepticism arose regarding the mindfulness intervention practice because the general public is perceived to harbour negative preconceptions toward it.

“Because I believe many people think it's (mindfulness) hocus-pocus... But I think CBT works if used in combination with returning to work.” [Individual interview 10]

mWorks was thought to *enable a proactive rehabilitation process*, where service users could be intercepted before a relapse. mWorks facilitated preventive and early interventions. These attributes were explained as important advantages of digital solutions, and made participants consider mWorks as a potentially effective RTW solution.

### Self-efficacy

Participants were *confident in their ability to use digital platforms* as a tool for RTW. They attributed their confidence to feeling comfortable with handling mobile devices in their everyday life. Participants explained that such social media is an integral part of smartphone usage. However, the use of additional applications such as mWorks on their smartphones could inhibit participant self-efficacy because the use of too many digital solutions can *increase the stress level* of an individual. They feared that their confidence in performing the required tasks in mWorks would be inhibited. They noted that smartphones were distracting because they are always prompting for attention. Participants explained the need to “take a break” from the phone. One participant explained:

“The mobile device has also become stressful, because as soon as I open it, there would be SMS and stuff … So, that is why I had to put it away for a while.” [Individual interview 7]

Another participant suggested that it was important to be able to turn of notifications or adjust notifications to the individual’s liking.

“Only the most essential should reach you. If Facebook and such things, notices should be on, … it can ruin a lot. You get distracted.” [Individual interview 3]

Participants perceived that the acceptability of digital solutions were especially *dependent on age and earlier digital experiences*. Older individuals were not believed to be as confident as the younger users, but could make up for it if they had prior experience of using digital tools on a more frequent basis.

## Discussion

The present study demonstrates that the expectations of mWorks as a digital RTW solutions are acceptable to service users with CMD. In essence, acceptability was present when mWorks focused on producing a positive affective attitude and fostering a stress and judgment free environment, where users can progress toward RTW to in a safe space. To increase perceived effectiveness, a digital solution needs to be designed to complement traditional RTW services and reflect an integrated and recovery-oriented approach. This contrasts to the stepwise, diagnosis and deficit-oriented approach that was deemed problematic. Attending to the perceived burden of usage is critical since this is related to motivational difficulties and cognitive strain among persons with CMD. Simultaneous user autonomy on approach and use of mWorks is necessary to avoid increased stress levels that risk reducing self-efficacy, and thus negatively impacting engagement levels.

Although there is ambiguity about the importance of human support for acceptance of digital solutions [26, 27], our findings indicate that the service user needs to be given the opportunity to direct the level of human interaction. Service users value the potential to complement traditional RTW services with access to fast and reliable human support, as well as the opportunity to progress towards RTW in a safe space with minimal amount of human interaction. That service users deem ordinary contact with RTW actors as too stressful has been observed elsewhere [44], and it is therefore crucial to consider the type, frequency, and duration of human support in order to optimize the delivery format [29]. This is imperative because the removal of human support might jeopardize positive effects on outcomes and the greater retention noted in previous research [28, 45, 46]. A way forward may be to use AI-directed chatbots that can serve to mimic human support and increase engagement and attrition rates of digital solutions [44]. In a recent trial it was concluded that these AI-directed conversational agents appear to be an engaging and effective way to deliver CBT for persons with CMD [47]. If similar effects can be derived for digital RTW solutions remains a subject for future research prospect.

The importance for service users to experience hope, power, and meet professionals who apply a person-centred and holistic approach during the RTW process are demonstrated to be critical RTW factors for persons with CMD [17]. Our findings suggest that mWorks must involve the entire RTW process to be perceived as effective. Service users described frustrations about traditional RTW services lack of sustainable support, and absence of a holistic approach throughout their RTW process. This fragmented process is hard to manage, and produces a sense of insecurity. Incorporation of important constituent elements in the RTW process through a digital solution can address this problem. One such element is inclusion of strategies to increase well-being and mental health, such as digital cognitive strategies, that has demonstrated to improve such outcomes [48, 49]. Another important element is the ability to plan and strategize RTW actions, as this is an important cornerstone in supported employment interventions [15, 16]. Thus, mWorks needs to incorporate a broad range of content in order to encompass the entire RTW process, when returning to and remaining at work, and be perceived as effective, and thereby acceptable.

mWorks was considered ethically acceptable if service users had the opportunity to control how to use and approach the digital solution. Research on developing digital solutions for health-related behavioural change indicates that offering too many choices or complete navigational control can be overwhelming and result in lower use. This points out the tension between supporting user autonomy and clear guidance on how to best engage with the intervention to change behaviours [36]. The more choices and actions service users are exposed to, the more cognitive strain is increased, and this could negatively affect acceptability. Indeed, too much freedom of use can be perceived as burdensome and result in lower levels of engagement compared to exposure to a coherent presentation of essential intervention components [50, 51]. The need to find a balance between dichotomies such as clear guidance or complete freedom in use of digital interventions has been noted elsewhere. Research is vital during the development phase in order to establish what services users prefer to do on their own and when clear directives are needed [32, 36]. Further research should investigate which activities users prefer to do without those activities becoming burdensome, and how to provide a system that allows independence and control.

Research has shown that symptom severity in depression is associated with lower levels of engagement [35]. This may constitute an acceptability barrier and impede engagement with mWorks if not addressed during development. Engagement barriers are one of the bigger barriers for implementation of digital innovations in European health care systems [46], since the average user spends 5 min or less on learning a digital solution [52]. Consideration of factors that ameliorate user deficits in engagement is crucial and could improve retention during digital RTW solutions. Future research on adequate strategies to increase motivation and provide users with tasks they find feasible and meaningful is needed. Positive feedback should be considered during development, as it can be a powerful tool to enhance motivation but must fit the needs of service users. RTW professionals have an important role when delivering mWorks. They have the opportunity to discuss and tailor feedback according to service user needs and preferences, in a way that is not possible in a digital context [34]. Similar to previous research [34], our findings further instantiate that service users’ fluctuation in symptom severity, and thus perceived burden, is a prominent acceptability barrier. This indicates that service users may be less likely to interact with cognitively demanding tools and that variability in symptoms needs to be accounted for in the development of digital solutions. Moreover, future research inquiries should investigate how digital solutions can account for this variability.

### Methodological considerations

The theoretical framework for acceptability provided a useful model as the accompanying components analyse specific aspects of the data corpus that are associated with service user perceived acceptability of a digital RTW solution. An unclear understanding of acceptability in previous literature resulted in an inadequately robust research corpus, and that interventions often fail to be embedded in practice [20, 53]. This situation reduced transferability of research findings, which the TFA framework can help to remedy. Co-production has been stressed as a main important factor to understand, reach and engage users that are going to use the digital solution in practice [45, 54, 55]. Thus, investigating acceptability prior to participation of mWorks was essential to increase the understanding of how the digital solution could be aligned with the users’ perspectives of acceptability in order to be integrated into practice. However, some constructs were challenging to employ in this setting because the intervention was in a formative stage, and intervention components were not fully determined. For example, it was not clear how the construct *intervention coherence* could help anticipate acceptability. Consequently, explaining the overarching intervention components to participants was essential. The framework would benefit from added clarification and development on how to analyse anticipated acceptability when the intervention still is in a formative stage.

Qualitative methods do not provide generalizable evidence. Therefore, generalized claims should be made with caution and finding herein might not be generalized to other contexts. However, the use of TFA and COREQ has improved research description and quality. In that sense, transferability becomes possible for readers, who can make own inferences [56]. Findings may further be interpreted as relevant to inform future development of the mWorks.

Although purposive snowball sampling was used since it yielded information-rich participants of a hard-to-find target group, the participants might not be representative of the entire group of persons with CMD and RTW experience. Although data saturation appeared to be met, with no additional insights arising in the final interview, it is possible that a wider range of participants (such as inclusion of younger people) would have provided additional aspects of acceptability. Future research should investigate needs and preferences of these subgroups to uncover all features of acceptability.

The authors have been or are currently involved in the development of mWorks and have preconceptions of what would be acceptable to the service users. This might have contributed to bias in the interpretation of the data. However, the analyses were conducted within a multidisciplinary research team with expertise in their respective research fields, including public health (PE), digital development and participatory research (PS), and research on SE and critical factors for RTW, and implementation (UB). This mixture of perspectives may have minimized personal biases and helped to ensure credibility of the findings. In addition, the deductive analysis according to the TFA model provided researchers with a common understanding of acceptability, which helps to mitigate biases, improves transferability, and thereby increases the trustworthiness of our study. Participants did not get the opportunity to member check our findings, which is a limitation of this study.

## Conclusions

This study sheds much-needed light on the acceptability of mWorks, and will help to inform future development of digital RTW interventions that are engaging and appealing to service users with CMD who are on sick leave. To create a positive user experience was addressed as vital. This entailed providing a safe digital space and a stress and judgment-free environment where service users have an opportunity to progress toward RTW. Perceived effectiveness was linked to the ability of mWorks to complement traditional RTW services with access to the entire RTW process according to user needs and resources. Participants found this a desirable departure from a diagnosis and deficit-oriented approach. Reducing the cognitive burden was perceived as critical for acceptability. High cognitive burden can jeopardize service user self-efficacy and negatively impact engagement levels. Future research should more fully explore perceived burden in order to understand the balance between user autonomy and other support components in digital solutions.

## Supplementary Information

**Additional file 1.**

## Data Availability

The interview guide is provided as Supplementary file 1. Original quotations from the data corpus are used to support the analysis procedure. A substantive, pseudo-anonymous collection of extracts from each participant interview is available from the corresponding author upon reasonable request.

## Abbreviations

CBT: Cognitive behavioural therapy
CMD: Common mental disorder
iCBT: Internet cognitive behavioural therapy
MRC: Medical Research Council
PES: Public Employment Service
RTW: Return to work
SIA: Social Insurance Agency
SMD: Severe mental disorders
TFA: Theoretical Framework of Acceptability
