# Supplementary material for: Acceptability of a digital return-to-work intervention for common mental disorders: a qualitative study on service user perspectives
Source: BMC Psychiatry. 2021 Aug 3;21:384. doi: 10.1186/s12888-021-03386-w (PMC8336332; doi:10.1186/s12888-021-03386-w)
Supplement: Supplementary file 1 — Additional file 1. [file 12888_2021_3386_MOESM1_ESM.docx]

Interviewguide for poeple with experience of common mental dissorder and sickleave

The purpose of the research project "mWorks-A mobile support back to work" is to develop and evaluate a digital tool to facilitate return to work and rehabilitation for people on sick leave with mental illness (depression, bipolar disorder and anxiety).

| **Main question** | **Probing question** |
| --- | --- |
| 1. What is your experience of using digital solutions such as the internet and mobile phone applications in connection with returning to work? | 1. Can you give examples of occasions when you have sought information, received support, or used strategies via the internet or your mobile phone to support return to work or during vocational rehabilitation? 2. If not, in what ways could you consider using a tool like mWorks? |
| **Perceptions of delivered intervention**   1. What do you think mWorks should contain to highlight your needs and preferences? | 1. How could mWorks make it easier for you when you start working again? |
| **Facilitating factors**   1. What opportunities and facilitation factors do you see for a mobile application to be successful for returning to work? | 1. What do you think would be a sign of success for such a digital aid? 2. If you were to look back, in five years, what would be proof of mWorks success? |
| **Barrier factors**   1. What obstacles do you see for such a digital mobile device to be successful? | 1. Give examples of support in connection with return to work that would have worked less well? 2. Explain why that type of support would not work in certain situations |
| **Future involvement**   1. Based on your situation, would you like to remain involved in the mWorks project? | 1. Under what circumstances could you imagine remaining active in the mWorks project? |
| **Snowball sampling**   1. Do you know suggestions on who whould be of intrest to interview? | 1. Do you know anyone with experience of return to work and sick leave due to depression, bipolar disorder, or anxiety we can talk to? |
